# The Utilization of Shape Memory Alloy as a Reinforcing Material in Building Structures: A Review

**DOI:** 10.3390/ma17112634

**Published:** 2024-05-29

**Authors:** Lidan Xu, Miaomiao Zhu, Jitao Zhao, Ming Chen, Mingfang Shi

**Affiliations:** 1 School of Civil Engineering, Inner Mongolia University of Science and Technology, Baotou 014010, China; 2 Intelligent Construction and Operation Engineering Research Center, Inner Mongolia University of Science and Technology, Baotou 014010, China; 3 Inner Mongolia Key Laboratory of Safety and Durability for Civil Engineering, Baotou 014010, China; 4 School of Civil and Architecture Engineering, Panzhihua University, Panzhihua 617000, China

**Keywords:** shape memory alloy (SMA), shape memory effect (SME), superelasticity (SE), reinforcement and repair, isolation and shock absorption

## Abstract

Shape memory alloy (SMA), a type of smart material, is widely used in the design of reinforcement and repair, isolation, and shock absorption of building structures because of its outstanding characteristics, such as the shape memory effect (SME), superelasticity (SE), and high damping. It not only improves the bearing capacity, ductility, and mechanical properties of the structural components of buildings but can also effectively slow down the strong response of engineering structures under the effect of an earthquake. It plays a key role in energy dissipation and shock absorption as well as sustainable development. To promote the application of SMA in building structures, this paper summarizes the research on the use of SMA as a reinforcing material in building structures, including work related to SMA material characteristics and types, SMA-reinforced structural components, and SMA isolation devices. In addition, the shortcomings of SMA applications in building structures are analyzed, and valuable suggestions for future research methods are put forward. SMA has been applied to engineering practice in the form of embedded and external reinforcement, which shows that it has broad application prospects in future buildings.

## 1. Introduction

The number of construction projects has increased with the rapid development of the economy and the continuous advancement of urbanization, and the safety of these projects has become a top priority [1,2,3]. With the development and updating of regulations and standards, many buildings, due to inherent problems such as design and construction methods, do not meet the current regulatory requirements. They are also affected by environmental and natural disasters (such as floods, earthquakes, typhoons, and fires), which reduce the strength of reinforced concrete (RC) structural components and cause damage and destruction, resulting in a considerable attenuation of their resistance and severe security hazards [4,5,6,7]. According to the “Uniform standard for design of civil buildings” [8], many buildings have reached the end of their service life, and there are significant challenges in ensuring the safety and durability of RC components. The reinforcement and transformation of existing, old buildings to meet people’s needs represent an alternative to demolition or reconstruction. At the same time, the tense situation caused by the continuous advancement of urbanization of land use has become increasingly prominent. To ensure the high quality and effective application of land resources, it is necessary to proactively plan in advance. It is urgent to scientifically evaluate the damage law and degree of existing buildings and carry out maintenance and reinforcement to delay the process of structural damage and prolong the service life of buildings. The existing data show that China is simultaneously in the stages of construction and maintenance and renovation, and developed countries such as the United States and Japan invest equally in strengthening and renovating existing buildings as in constructing new buildings. Therefore, it is of great significance to repair and reinforce existing buildings based on various damage factors and damage degrees.

**Figure 1:**
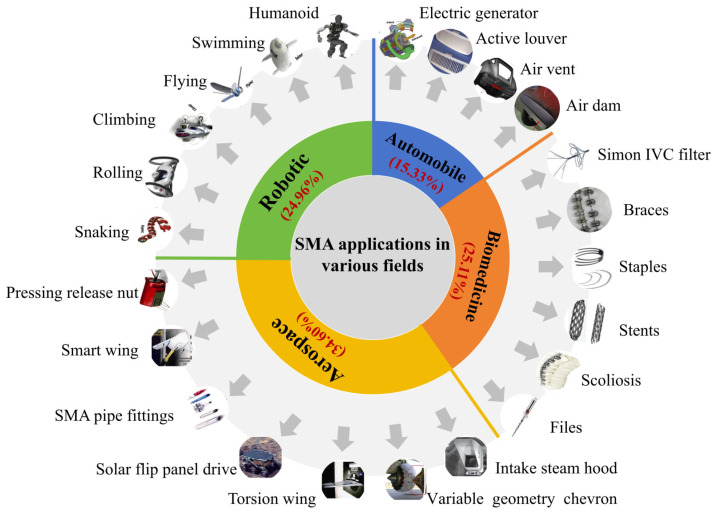
Applications of SMA in various fields.

**Figure 2:**
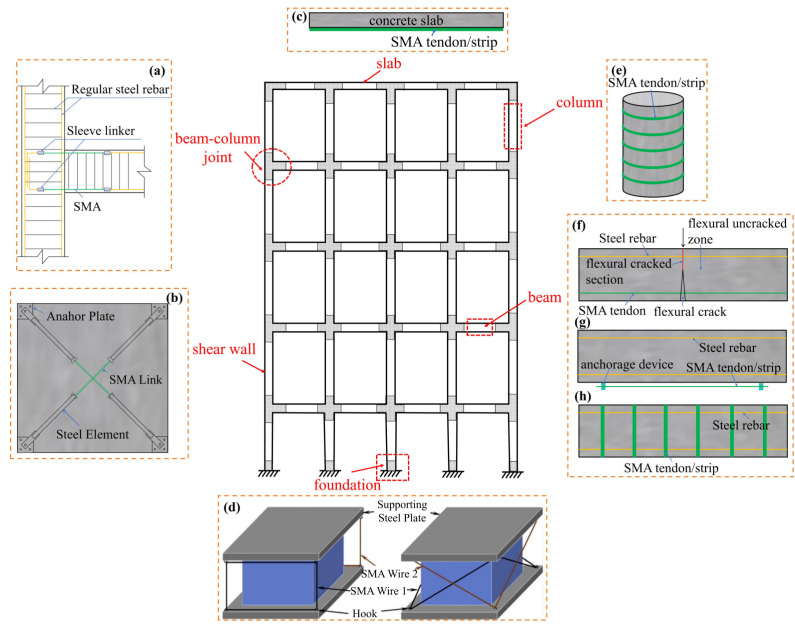
Applications of SMA in building structures: (**a**) SMA-reinforced beam–column joints; (**b**) SMA-reinforced shear walls; (**c**) SMA-reinforced slabs; (**d**) SMA damping energy dissipation components; (**e**) SMA-reinforced columns; (**f**) self-healing of SMA beams; (**g**) SMA bending-reinforced beams; (**h**) SMA shear-reinforced beams.

**Figure 3:**
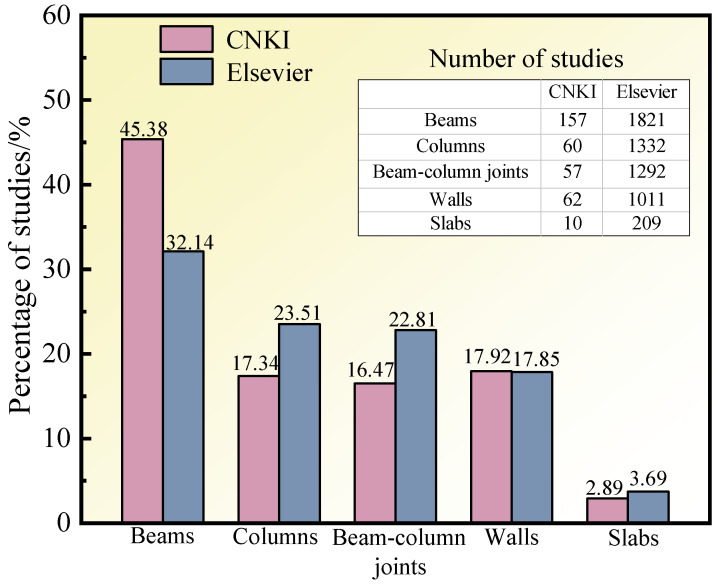
Research on the application of SMA in various components of building structures from 2003 to 2023.

Common reinforcement methods include the increasing section reinforcement method [9], cladding steel reinforcement method [10,11], and prestressed reinforcement method [12,13]. Although these approaches can improve the mechanical performance of RC structural members to varying degrees, they do not comprehensively account for economy and efficiency and have their defects. To solve these problems, it is necessary to use more advanced process methods and new materials to improve the reinforcement technology and obtain a simple and feasible reinforcement method. Shape memory alloy (SMA), as a smart material, has many desirable properties such as the shape memory effect (SME), superelasticity (SE), high energy consumption, and biocompatibility. It has been widely used in the automobile [14,15,16], aerospace [17,18,19,20], robotics [21,22], and biomedical [23,24,25] fields, as shown in Figure 1. SMA has a higher energy density, higher driving frequency, larger output stress and strain, and higher fatigue resistance than other advanced materials such as magnetorheological, piezoelectric, and viscoelastic materials. Its comprehensive performance is superior, and it has wide application potential in engineering, giving it broad appeal. With the progress of research and technology, SMA has gradually been applied in the field of building structures, as shown in Figure 2. Its advantages are mainly reflected in three aspects: ① The SE of SMA endows the structure with a good energy dissipation capacity and self-resetting capacity, meaning that cracks and displacements in the structure can easily be suppressed. ② Due to its SME, SMA can produce a large driving force, which can apply prestress to the structure, increase the cracking load, and improve the overall performance of the structure. ③ Reasonable use of high damping characteristics of SMA can lead to significant energy consumption. The structural vibration response can thus be reduced, and shock absorption can be achieved. Figure 3 shows the literature survey results on the application of SMA to various components of building structures from 2003 to 2023 obtained through the official China National Knowledge Infrastructure (CNKI) and Elsevier websites. The research on SMA applications in building structures focuses on beam components (38.76% on average), and the average proportions of research on column components, beam–column joints, and shear walls are 20.43%, 19.64%, and 17.88%, respectively. There is very little research on slab components (3.29% on average).

This paper summarizes the application of the unique properties of different forms (rods, wires, plates, and strips) of SMA in building structures, and Appendix A summarizes the application of SMA in various building structural components (beams, columns, beam–column joints, shear walls, and slabs) in the past decade. It is found from the table that SMA has been widely used in the reinforcement and restoration of building structures. Especially in the past five years, Fe-SMA has attracted the attention of researchers because of its relatively low price and made the application scenarios of SMA more extensive. In this paper, the characteristics, types, and mechanism of SMA are expounded. Then, starting from the application of SMA in building structures, the role of SMA in different structural components of building (beams, columns, beam–column joints, walls, and plates, etc.) is analyzed, and the shortcomings of existing research are outlined. Finally, combined with the application of SMA in practical engineering, the future research and development trend of SMA materials is analyzed to provide a reference for further research of SMA in building structures.

## 2. SMA Characteristics and Types

SMA has a development history of nearly 90 years. Swedish scientist Ölander [26] first discovered the phenomenon of martensite fluctuation with temperature variation when studying Au-Cd alloy in 1932, which was a prelude to the study of SMA materials. In the following 30 years, American scholars Chang et al. [27] and Rachikger [28] also observed similar phenomena in Au-Cd and Cu-Al-Ni alloys, respectively. In 1962, Buehler et al. [29] in the United States discovered memory behavior in Ni-Ti alloy, which was coined the SME and attracted wide attention from researchers. Since then, many scholars have studied the performance, microstructure, and types of SMA, laying the foundation for its application.

### 2.1. SMA Characteristics

#### 2.1.1. SMA Phase Transition

**Figure 4:**
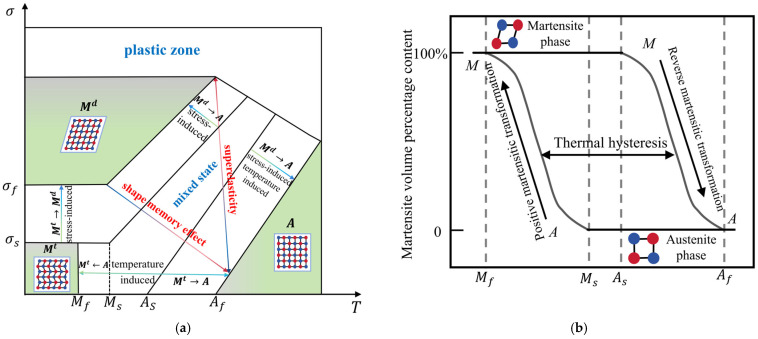
SMA phase transition process: (**a**) schematic diagram of shape memory alloy microstructure phase transition; (**b**) martensite volume percentage and temperature diagram.

SMA has two different crystal structure states: One exists in the form of the martensite phase at lower temperatures, with characteristics of low hardness, easy deformation, and stability at low temperatures. The other is in the form of the austenite phase or parent phase at higher temperatures and has the characteristics of high hardness, low deformation, and stability at high temperatures. Moreover, the transformation between the austenite phase and the martensite phase can be realized by changing the temperature or applying external forces [30,31]. SMA has four characteristic phase transition temperatures, which are martensite start (M_f_), martensite finish (M_s_), austenite start (A_s_), and austenite finish (A_f_), among which M_f_ < M_s_ < A_s_ < A_f_. At room temperature, SMA generally exists in the parent phase, namely the austenite phase. When the ambient temperature of SMA is below M_s_, the austenite contained within will begin to transform into martensite. The transformation process is not declared complete until the ambient temperature is below M_f_, at which point its martensite content is 100%. When the ambient temperature of SMA is heated to A_s_, martensite will transform into austenite, and the process will not be fully completed until its temperature rises to Af [32,33,34], as shown in Figure 4.

The phase transition behavior of NiTi-based SMA is significantly affected by the chemical composition and microstructure of the alloy. The phase transition path of solid solution binary NiTi alloy is B2 (parent phase, cubic crystal structure) → B19′ (martensitic phase, monoclinic crystal structure). The change in microstructure [35,36] (grain size, precipitated phase, dislocation, etc.) can cause the R phase to appear in the phase transition path of NiTi SMA. The R phase is the pre-phase transition product of the alloy before the martensitic transition and the R phase transition is the same as the martensitic phase transition. In the process of deformation, the surface of the phase can undergo a convex elastic change, but the deformation variable is much smaller than that of the martensitic phase transition, accounting for only about 1/10 [37]. Doping of many elements also changed the phase transition path of NiTi alloy; for example, the doping of the Fe element caused the appearance of the R phase [38], and the doping of the Cu element caused the appearance of the B19 phase (martensite phase, rhomboidal structure) [39]. Nb, Fe, Co, Cr, V, Mn, A1 [40,41], and other elements reduced the martensitic phase transition point of NiTi alloy, while Pt, Pd, Hf, Ta, Zr, Au, Cu [42,43,44], and other elements increased it.

#### 2.1.2. SME

**Figure 5:**
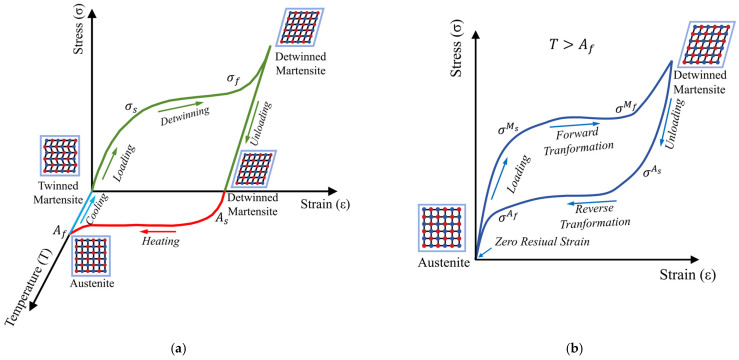
Schematic diagram of SMA characteristics: (**a**) SME; (**b**) SE.

The SME refers to the plastic deformation recovery performance of SMA in a low-temperature martensitic state, where the material’s plastic deformation is restored after an increase in ambient temperature due to a certain limit of unloading and irreversible plastic deformation caused by external forces [45], as shown in Figure 5a. The SME is essentially caused by martensitic transformation inside the alloy. In short, the SMA is first heated above the temperature T > A_f_ to shape, and the interior of the specimen is mostly austenite phase. It is then cooled below the temperature T < M_f_ and unloaded after loading to achieve residual deformation. When the deformed specimen is reheated to the above A_f_ level, the specimen will return to the shape before the deformation [46,47].

#### 2.1.3. SE

SMA is not only known for its SME but also for its excellent SE and has gained widespread attention. The strain of SMA in the austenitic phase state is much higher than the elastic limit strain of the alloy, and the phenomenon whereby the deformation can automatically recover after unloading is called SE, as shown in Figure 5b. The essence of SE is the internal friction phenomenon in the process of stress-induced martensitic transformation and martensitic reverse transformation. Specifically, SMA maintains an austenitic phase transition at room temperature, which generally depends on the alloy composition and the resulting phase transition temperature. At this time, multiple martensitic variants tend to a single variant in the direction most conducive toward deformation, leading to the deformation of the alloy. However, this type of martensite remains stable only under external stress. When the alloy is unloaded, the martensitic instability immediately undergoes a reverse phase transformation back to the parent phase, and the shape of the alloy also returns to its original state [48,49,50].

#### 2.1.4. High Damping

Damping capacity is used to describe the ability of a material to block energy in the process of structural mechanical vibration or wave propagation through deformation and gradually dissipate it. Damping performance is an important index used to monitor the energy consumption, vibration, and fatigue fracture of materials. SMA has good damping performance due to the self-coordination ability of martensitic transition, the formation of various interfaces (phase interfaces, twin interfaces, variant interfaces, etc.), and the relative movement of the interfaces (hysteretic elastic migration) absorbing a large amount of energy during the transformation process [51]. Studies have shown that the damping magnitude of SMA is the mixed coexistence state of martensitic phase and austenite phase > martensite phase state > complete austenite phase state [52].

### 2.2. Types of SMA

**Figure 6:**
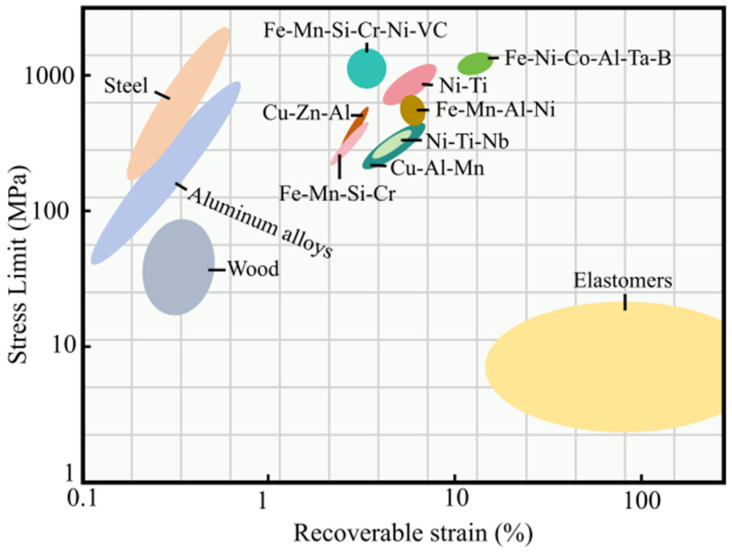
Comparison between common building materials and different types of SMA (adapted from [60]).

More than 100 types of SMA have been discovered to date, among which the most widely used are three types of SMA based on nickel titanium (NiTi-SMA), copper (Cu-SMA) and iron (Fe-SMA). Ni-Ti alloy is the earliest studied, and the production process has reached a mature stage of commercialization. It has the advantages of large recoverable strain/stress, strong adaptive ability, high tensile strength, excellent corrosion resistance, good fatigue resistance, and ease of coupling with other substrates, but its high price limits its development [53,54]. Cu-based alloy has a low price (about 1/10 of Ni-Ti alloy), and its phase transition has an excellent SE effect, but its recovery stress is low. It also has coarse grains, easy fracture of grain boundaries, and poor phase transition stability, resulting in slow progress [55]. Fe-based alloy has attracted increasing attention because of its comparable stress and low cost compared with Ni-Ti alloy, but its poor corrosion resistance and unstable performance mean that it is not as widely used as Ni-Ti alloy [56,57]. Figure 6 shows the ultimate stress and recovery strain of commonly used building materials and different types of SMA. Steel and aluminum are displayed next to the longitudinal axis, indicating that they have high strength but relatively low recovery strain. The elastomer is located next to the horizontal axis, indicating that this type of material has relatively low strength, but it is more likely to restore its shape after unloading. Most of the SMAs are concentrated in the same location, demonstrating that the strength and recoverability of SMA have a better balance, wherein the recovery strain of Ni-Ti alloy is relatively stable, and the recovery strain of Fe-Ni-Co-Al-Ta-B is higher [58,59,60].

## 3. The Application of SMA in Structural Components of Building

**Figure 7:**
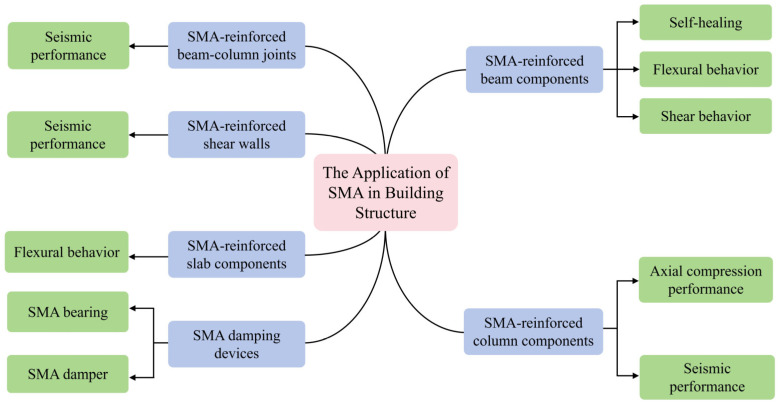
Application of SMA in various aspects of building structures.

Due to the characteristics of SMA such as the SME and SE, its application in building structures has been widely studied. Among these studies, a considerable amount of research has been conducted on the application of SMA-reinforced beams, columns, beam–column joint areas, shear walls, and slab components, as shown in Figure 7. The related applications and development of SMA will be introduced in the following section.

### 3.1. SMA-Reinforced Concrete Beams

Concrete beams are among the main load-bearing components in building structures and are widely used. The research on SMA used in beam components focuses on the self-healing, flexural, and shear performance of concrete beams. Therefore, the following three aspects are introduced.

#### 3.1.1. Self-Healing Performance

**Figure 8:**
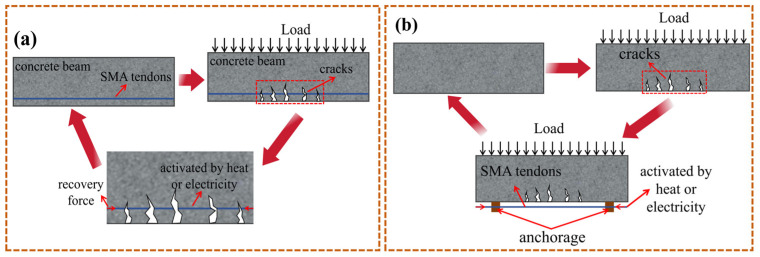
Two installation techniques for self-healing of SMA in concrete beams: (**a**) internal: replacement of tension reinforcement; (**b**) external: fixing with anchors in the tensile zone of the beam.

Concrete is a type of brittle material that inevitably exhibits many different forms and degrees of cracks during the construction and use of concrete structures. This not only affects the appearance of the structure but also inhibits its normal function. The original deflection and cracks in SMA can be restored by utilizing the SME and recovery deformation to generate a large driving force. Figure 8 shows that there are two installation techniques for self-healing of SMA. One is to replace the tensile reinforcement of beams with SMA, and the other is to fix SMA in the tensile area of the beam with anchors. After the component is deformed or cracked under load conditions, the SMA is thermally excited. At this time, the restoring force generated by SMA can act on the structure to heal cracks in concrete beams.

**Figure 9:**
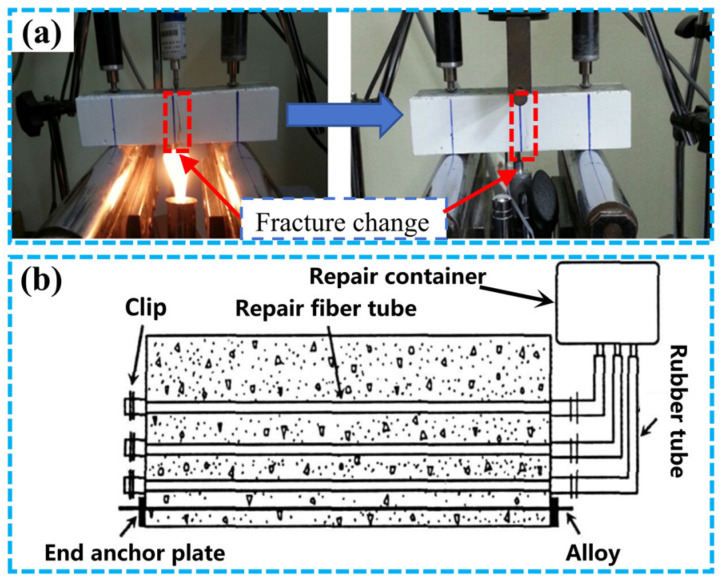
Repair performance of SMA concrete beams: (**a**) changes in cracks before and after repair of SMA beams [63]; (**b**) a smart concrete beam with a self-healing function [66].

Sakai et al. [61] used SE SMA to embed SMA wires in the austenite state in mortar beams for the three-point bending test. The results showed that the deformation range of the SMA mortar beam was more than seven times that of the reinforced mortar beam, and almost all of its cracks were closed after unloading. Lee et al. [62] and Choi et al. [63] later studied the effect of SMA reinforcement by replacing tensile steel bars on the self-healing performance of the beam. Choi et al. [63] performed thermal excitation of SMA with fire to close cracks, as shown in Figure 9a. Li et al. [64,65] proposed a new repair method: CFRP plate-SMA steel wire composite reinforcement of simply supported beams. Through experiments and finite element analysis, it was found that SMA reinforcement played an important role in reducing residual deformation and closing cracks.

In addition, Kuang and Ou [66] combined SE SMA with the bonding properties of repair adhesives to create self-healing concrete beams. Through static load tests, it was found that the SE of SMA could drive the repair of cracks. The brittle fibers containing the adhesive broke and released the adhesive into the cracks to complete the repair. However, due to the complexity of this structure, the arrangement of SMA filament and repair fibers was difficult, which limited the further application of this method. The specific device is shown in Figure 9b. Bonilla et al. [67] combined SMA with microcapsules to repair cracks in concrete and used crack monitoring and energy-dispersive X-ray spectroscopy to evaluate the healing of cracks over time and characterize the healing components in the crack region. The results showed that the combination of the two approaches could lead to better repair of cracks in concrete.

In addition to embedding alloy wire into a beam, Xue et al. [68] fixed NiTi-SMA wire as external prestressed tendons outside an RC beam, stopped loading after reaching a certain load value, and then performed thermal excitation on the alloy wire, so that the deflection of the concrete beam in the early loading process could be restored to a certain extent. Yang et al. [69] and Sun et al. [70,71] compared the repair of beam cracks using two methods of embedded SMA and externally installed SMA and studied the effect of unbonded length on the self-healing ability of embedded SMA wires. The results revealed that the cracks could be repaired effectively using both methods. The longer the unbonded length of embedded SMA, the better the crack repair.

In summary, SMA can indeed play an effective role in the repair of concrete cracks. However, the size of the SMA concrete beam components studied is relatively small, and this effect has not been considered. The size of the components may affect the effectiveness of the alloy in practical engineering. Therefore, more research should be conducted on full-scale beams in practical engineering. Regarding the repair problems of existing projects, the external installation of SMA is more due to the internal setting of SMA. However, the anchorage and excitation methods used need to be further optimized. Reliable and applicable prestressed anchorage and excitation methods have been developed to ensure the stability of prestress and have provided a stronger theoretical basis and technical support for the application of SMA in crack repair of concrete components.

#### 3.1.2. Flexural Performance

In this type of research, the pre-deformed SMA is usually installed and fixed in the tensile zone of the beam, and thermal excitation is immediately applied to it. The recovery force generated by the SMA causes the structure to prestress, thereby improving its bending capacity. SMA can be used to strengthen concrete beams via near-surface mounting (NSM), embedding in the shotcrete layer, external fixation, and internal setting. The research on using these four installation techniques used to strengthen concrete beams to improve their flexural performance is described below.

**Figure 10:**
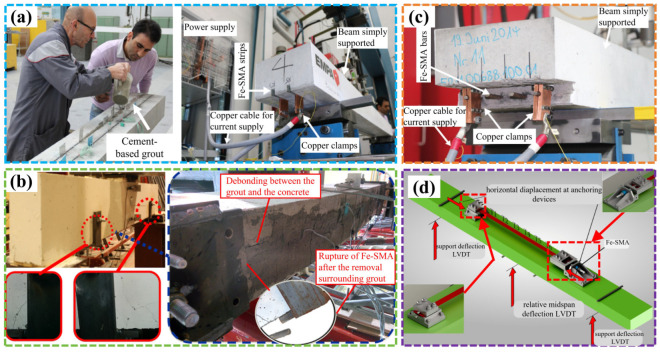
Flexural properties of SMA-strengthened RC beam: (**a**) NSM of Fe-SMA plate [72]; (**b**) NSM of Fe-SMA bars [75,76]; (**c**) Fe-SMA bars installed in the new shotcrete layer at the bottom of the beam [77]; (**d**) external mechanical anchoring of Fe-SMA strips at the bottom of concrete beams [80].

Most researchers in this field have adopted the method of NSM to achieve prestressed reinforcement of concrete beams and verified the effectiveness of this approach. In this method, grooves are first carved on the concrete protective layer, SMA rebars/slats are placed in the grooves, and then epoxy resin or cement mortar are used to fill in the grooves. Shahverdi et al. [72] and Hong et al. [73] embedded Fe-SMA plates in the protective layers of RC beams and then thermally stimulated the SMA to generate prestress. The flexural performance of the prestressed concrete beams was tested, as shown in Figure 10a. It was found that prestressed reinforcement with SMA could obviously increase the cracking load, improve the fatigue resistance, and reduce the crack width and mid-span deflection of concrete beams. Then, Yeon et al. [74] used OpenSees software (https://opensees.berkeley.edu/) to conduct finite element modeling and analysis of the reference [72], verified the validity of the finite element model, and studied the influence of concrete strength, level of reinforcement, and number of Fe-SMA strips on the bending behavior of RC beams. In addition, Canadian scholars Rojob and El-Hacha [75,76] conducted experimental studies on the flexural properties of prestressed RC beams strengthened with NSM Fe-SMA reinforcement in long-term freeze–thaw cycles and under fatigue load conditions, as shown in Figure 10b. The results showed that using NSM Fe-SMA reinforcement significantly improved the performance of RC beams under service load conditions.

Shahverdi et al. [77] also conducted a bending study on beams reinforced with pre-embedded SMA steel bars in a sprayed concrete layer, as shown in Figure 10c. The results revealed that the prestressed Fe-SMA rebars significantly improved the performance of the normal use stage. It was feasible to embed ribbed Fe-SMA bars in the newly laid shotcrete layer on the bottom of the reinforced concrete beam, and the reinforcement effect was good. Dolatabadi et al. [78] used ABAQUS to conduct numerical studies on RC beams reinforced by Fe-SMA reinforcement and shotcrete in reference [77]. Michels et al. [79] and Strieder et al. [80] also studied the bending performance of concrete beams reinforced with external Fe-SMA strips. Michels et al. [79] used shooting nails to anchor SMA strips and concrete, while Strieder et al. [80] developed a special clamping device to anchor SMA strips and concrete, as shown in Figure 10d. The results showed that there was no damage to the anchorage at the end of the Fe-SMA strip during the loading process. The cracking load, yield load, and ultimate load of the reinforced beam were significantly increased. Due to the effect of prestressing SMA, the development of cracks was also improved.

Some scholars have studied the influence of SMA as a longitudinal reinforcement on the flexural performance of RC beams. Abdulridha et al. [81] studied the mechanical properties of concrete beams with SE SMA bars replacing ordinary steel bars in the critical region (pure bend section of the beam) under monotonic and cyclic loads. The results showed that the existence of SMA bars improved the energy dissipation capacity of beams under cyclic loading conditions. Hong et al. [82] studied the flexural performance of concrete beams with Fe-SMA steel bars of different cross-sectional areas as tensile steel bars and found that the cracking load of concrete beams was significantly increased after SMA activation (by 47.6–113%).

In summary, SMA can improve the flexural bearing capacity and deformation capacity of RC beams regardless of the installation technology used. In existing studies, SMA is usually excited by electric heating to apply prestress, but this method requires a large electric current, which has security risks and low economic practicability. Secondly, in practical engineering, beams are often reinforced after certain damage occurs, and the existing studies are mainly related to the flexural resistance of intact beams; so, it is necessary to explore the performance of test beams under secondary loading conditions.

#### 3.1.3. Shear Performance

Concrete beams under load will experience bending and shear failure. Compared with bending failure, shear failure is less anticipated and more obvious and can cause large-scale casualties and economic losses. By utilizing the SME characteristics of SMA, prestress can be generated without tension on site, realizing active shear reinforcement of concrete beams.

**Figure 11:**
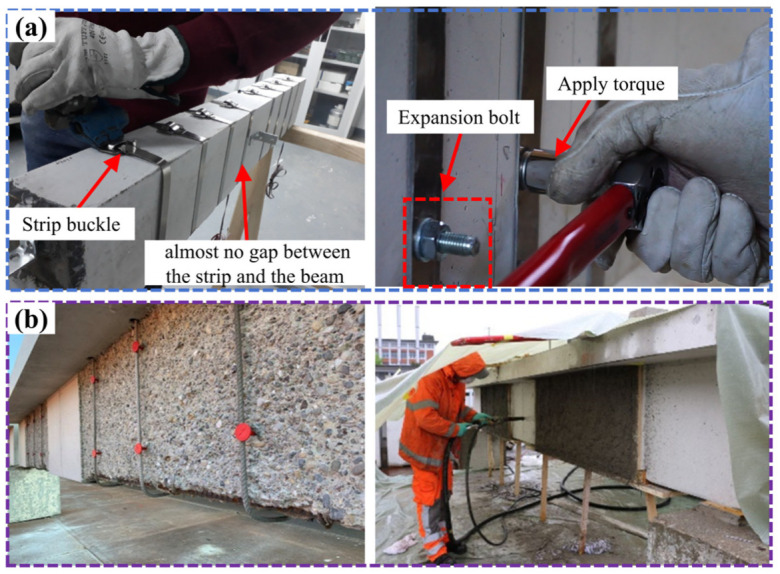
SMA shear-strengthened RC beam: (**a**) anchorage method [83,84]; (**b**) stirrups embedded with sprayed mortar [87].

Other researchers [83,84,85] have produced closed hoops with Fe-SMA strips for shear reinforcement of concrete beams and used industrial buckles or screws to anchor the strips, as shown in Figure 11a. Although there was still a small gap between the strip and the beam after anchoring, the gap disappeared after energized excitation. It was found that the SMA excitation not only improved the shear strength of the concrete beam but also effectively alleviated the development of cracks. Ruiz-Pinilla et al. [86] later proposed a constitutive model of Fe-SMA according to the Ramberg–Osgood model and simulated the experiments in reference [83] to verify the effectiveness of the model. Czaderski et al. [87] from the EMPA Laboratory in Switzerland combined U-shaped Fe-SMA bars with sprayed cement mortar to strengthen T-shaped concrete beams, as shown in Figure 11b. The results indicated that the shear resistance of RC T-beams was obviously improved under the effect of Fe-SMA stirrups and a new mortar layer. After the shear failure occurred, there was no interface failure between the new mortar layer and the original concrete, and the Fe-SMA stirrup did not fail.

The shear strengthening of RC beams using SE SMA was studied by Mas et al. [88], who investigated the effect of SE Ni-Ti SMA rectangular spirals on the performance of shear critical RC beams. In recent years, SMA has been used for shear reinforcement of RC beams in the form of internal stirrups. Ji et al. [89] and Hong et al. [90] studied the influence of Fe-SMA stirrup spacing and its effect shear performance and used finite element software for simulation and analysis. The results showed that SMA stirrups improved the shear strength and initial stiffness of the component and delayed the formation of cracks, verifying the effectiveness of the model.

In summary, SMA can indeed play an effective role in the shear reinforcement of concrete beams, but it requires high anchoring technology to ensure that the gap between SMA and concrete is as small as possible. Considering that the external enclosed hoop form of SMA does not meet the requirements to be applied to practical engineering, it is necessary to propose new reinforcement methods and anchoring systems and carry out research to find suitable alternatives.

### 3.2. SMA-Reinforced Concrete Columns

In building structures, concrete columns are the main load-bearing components, and they are more susceptible to the degradation of mechanical properties than other components. The exploration of effective repair and reinforcement of columns has become an urgent task. SMA materials have excellent damping properties and energy consumption capacity due to the self-coordination of martensitic phase transition and the various phase interfaces formed in martensitic, as well as the movement of the interfaces. Therefore, SMA can be used for external winding arrangements of RC columns in the form of wires or strips to enhance the axial compression and seismic performance of concrete column members.

#### 3.2.1. Axial Compression Performance

When SMA is wrapped on the surface of RC columns in the form of wires or strips, it will exert a restraining effect on the internal concrete. The ultimate strength and deformation ability of concrete columns can be improved comprehensively due to the strong lateral confinement ability and good deformation ability of SMA.

Andrawes et al. [91,92,93] and Choi et al. [94] almost simultaneously proposed a method of applying lateral confinement to concrete columns via thermally triggered prestressing of martensitic SMA wires. Choi et al. [95,96] conducted a comparative test on the axial compression properties of SMA-reinforced specimens and thin-walled steel tube reinforced specimens. The results showed that the failure strain of the SMA-wire-reinforced concrete column was significantly increased, up to 20%, and the energy dissipation performance of the column was greatly improved. With the same volume configuration ratio, the enhancement effect of SMA on the ultimate bearing capacity and failure deformation of concrete columns was better than that of thin-walled steel tube reinforcement. Subsequently, Tran et al. [97], Gholampour et al. [98], and Hong et al. [99] studied the effects of different factors on the axial compression performance of concrete columns strengthened by SMA, such as the amount of SMA, the level of prestress, and the form of confinement. The results demonstrated that SMA wire could enhance the axial bearing capacity and deformation properties of concrete columns. The ultimate bearing capacity of concrete columns also increased with the increase in the amount of SMA and the level of prestress. Chen et al. [100] and Suhail et al. [101] compared the axial compression performance of concrete columns confined by SMA and fiber-reinforced polymers/plastics (FRPs), while El-Hacha et al. [102] studied the compression performance of concrete columns reinforced by SMA wire under eccentric load. The results showed that the SMA confined columns exhibited higher ductility and residual strength than the confined FRP members.

The above studies analyzed the axial compression properties of SMA-strengthened concrete columns from the experimental perspective, while Chen et al. [103,104] and Abdelrahman et al. [105] further studied the performance of SMA active reinforcement from a theoretical viewpoint. Based on the plastic theory, the NiTiNb-SMA confined concrete constitutive model was proposed. This model can be utilized to predict and simulate the three-dimensional stress–strain behavior of NiTiNb-SMA confined concrete under monotonic and cyclic loads. In the in-depth study of SMA, Fe-SMA has attracted the attention of researchers because of its lower cost. Jeong et al. [106] compared the axial compression performance of concrete columns confined by Fe-SMA strips and carbon fiber-reinforced plastics (CFRPs), while Han et al. [107] investigated the influence of three variables (constraint types, number of FRP strip layers, and strip spacing) on their performance. The results showed that the bearing capacity and deformation capacity of concrete columns could be effectively improved by all three constraint types, but the Fe-SMA active constraint was more efficient. Meanwhile, the combined constraints obviously influenced the improvement of the mechanical properties of concrete columns. Han et al. [107] proposed a verification calculation method for predicting the peak compressive stress of these specimens, providing a reference for selecting appropriate concrete column reinforcement methods. Subsequently, Zerbe et al. [108] evaluated the effects of different parameters (the presence of internal reinforcement, initial confining pressure, and the ratio of external SMA confinement) on the axial compression performance of Fe-SMA strips confined concrete columns through experiments. In addition, an analytical method was proposed to simulate the axial load–deformation behavior of the active confinement column. Vieira et al. [109] used finite element software to simulate and analyze the experiment, verified the correctness of the proposed model, and showed that the model could also predict the compression performance of SMA confined concrete columns under eccentric loads.

**Figure 12:**
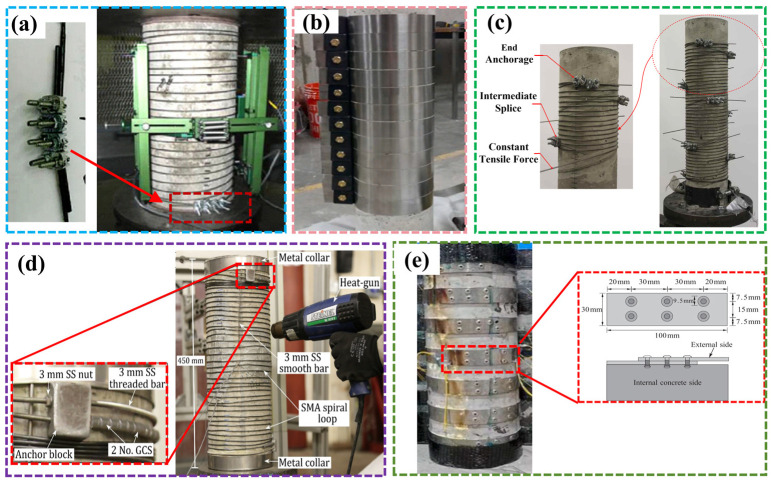
Anchorage methods of SMA-reinforced concrete column: (**a**) U-shaped clamp connection [92]; (**b**) drilling hole anchoring at both ends of the strip [108]; (**c**) U-shaped clamp anchoring fixed to the concrete column [102]; (**d**) 3 mm stainless steel threaded rod connection [101]; (**e**) rivet drilling [107].

To sum up, SMA can effectively enhance the axial compression performance of column members. Reasonable anchoring methods are crucial in this regard, Figure 12 lists the anchoring methods of SMA-reinforced columns in the literature. Secondly, the existing research mainly focuses on the axial compression performance of circular cross-section columns, while in practical engineering, the cross-section forms of columns are diverse and are mostly complex situations under eccentric or multiple loads. Therefore, the performance of concrete columns under complex stress conditions still requires further study.

#### 3.2.2. Seismic Performance

As the key bearing parts of building structures, RC columns have large axial compressive capacity and stiffness and often fail due to insufficient ductility. Brittle failure can easily occur due to insufficient deformation under the effect of an earthquake, which threatens the overall safety and reliability and even the collapse of the structure. Shin and Anderawes [110,111,112] conducted quasi-static tests on four 1/3-scaled RC columns and studied the effects of constraint methods and materials on the seismic performance of RC columns to determine the seismic ability of SMA-reinforced columns. The results showed that SMA active reinforcement columns had the highest degree of improvement in flexural ductility and energy dissipation capacity and the best control of concrete damage. In addition, the researchers [113] also produced spiral-shaped NiTiNb SMA to repair severely damaged RC columns. The repair time of each column was limited to 15 h, and quasi-static tests were performed on the columns within 24 h after the repair began. The results showed that the lateral strength of the two repaired RC columns was completely restored. This method significantly improved the lateral stiffness and ductility of earthquake-damaged RC columns.

In summary, few studies have been conducted on the seismic performance of SMA-reinforced columns in building structures. Through investigation, it was found that the research on the seismic performance of SMA columns focuses on the reinforcement of bridge pier columns using SMA in the form of longitudinal steel bars. Therefore, it is necessary to strengthen the research on this aspect using finite element simulation to achieve a more comprehensive understanding.

### 3.3. SMA-Reinforced Beam–Column Joints

The beam–column joint is the most essential and fragile part of the building structure and is responsible for distributing bending moment as well as transferring shear force and axial force. Many earthquake damage investigations have shown that beam–column joints are easily damaged under seismic force conditions and their failure will cause the destruction or even collapse of the whole structure. Therefore, improving the seismic performance of beam–column joints is particularly important for improving the seismic performance of the entire building. The beam–column joint area is enhanced using SMA to improve the energy dissipation and deformation recovery capacity of the structure under the effect of an earthquake.

**Figure 13:**
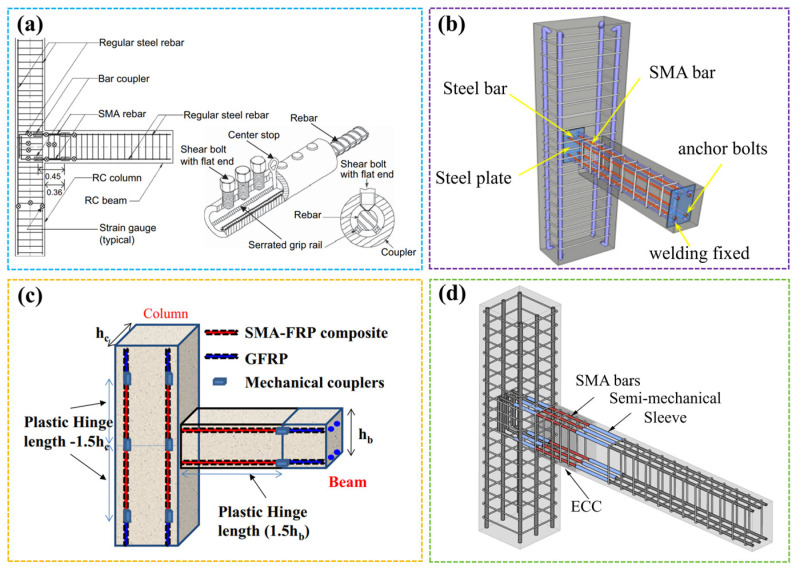
SMA-reinforced beam–column joints: (**a**) schematic diagram of the SMA joint [114,115]; (**b**) 3D sketch of the self-centering joint [120]; (**c**) schematic diagram of the SMA-FRP joint [121]; (**d**) diagram of the beam–column joint [125].

Researchers have carried out relevant work on the seismic performance of SMA concrete beam–column joints. Youssef et al. [114,115,116] and Naher et al. [117] buried SE SMA bars as longitudinal reinforcements of beams and conducted experimental research and finite element analysis, as shown in Figure 13a. The results showed that the SE SMA reduced the residual deformation of beam–column joints compared with ordinary beam–column joints and had good self-resetting ability, deformation ability, and plastic hinge rotation ability. Oudah and El-Hacha [118,119] later considered using SMA to reinforce the plastic hinge zone of concrete to enhance the self-centering capability and reduce the direct cost of materials. SMA reduced joint deformation and moved the plastic hinge area away from the cylinder.

Pei et al. [120] put forward a new self-centering joint using SE SMA rods and steel plates as core components in the core area of the node, as shown in Figure 13b. The new joint structure of SMA reinforcement and steel plates improved the bearing capacity of the joint, delayed the stiffness degradation, and improved the ductility and self-centering capacity of the joint. Zafar and Andrawes [121] studied a new fiber-reinforced polymer (FRP) embedded with SE SMA fibers, as shown in Figure 13c. The results indicated that the use of SMA-FRP bars in the plastic hinge zone significantly reduced the accumulation of permanent damage and residual deformation, thereby improving the overall performance of the frame under continuous earthquake disaster conditions.

Navarro-Gomez and Bonet [122] used SMA bars and ultra-high-performance concrete (UHPC) in key areas of the structure to improve the seismic performance of the structure. The results showed that the combination of SMA and UHPC reduced deformation. Qian et al. [123,124,125] studied the seismic performance of RC frame joints reinforced by SE SMA bars and ultra-high-toughness, fiber-reinforced, engineering-cement-based composite (ECC), as shown in Figure 13d. The results revealed that pouring ECC after the plastic hinge area of the beam–column joint significantly improved the failure mode and ductility of the joint. SMA-ECC-reinforced beam–column joints achieved better functional self-healing ability after earthquakes and exhibited better self-healing performance in crack healing and internal damage repair.

In summary, the research of SMA-reinforced beam–column joints mainly focuses on the analysis of their seismic performance. Most of the research methods are combined with experimental analysis and numerical simulation, but relevant theoretical analysis is lacking. The research on the connection between SMA bars and steel bars in the beam–column joint area is insufficient. In addition, from the overall point of view of the frame structure system, comparative analysis of the seismic performance of different types of joints (such as end joints and middle joints) is still relatively rare, and it is worth conducting more comprehensive discussions.

### 3.4. SMA-Reinforced Shear Walls and Concrete Slabs

Shear walls are the main lateral force-resistant components of high-rise structures in earthquake-fortified areas. Their performance is crucial to the seismic performance of the entire building structure. The traditional method is to rely on improving the stiffness and strength of the component itself, but this approach cannot solve the problem of excessive residual deformation and macroscopic cracks of the structure under strong earthquake conditions. The SE of SMA can provide better energy dissipation capacity for shear walls and enhance their self-resetting ability and self-healing ability to improve the seismic performance and durability of the shear wall structure.

**Figure 14:**
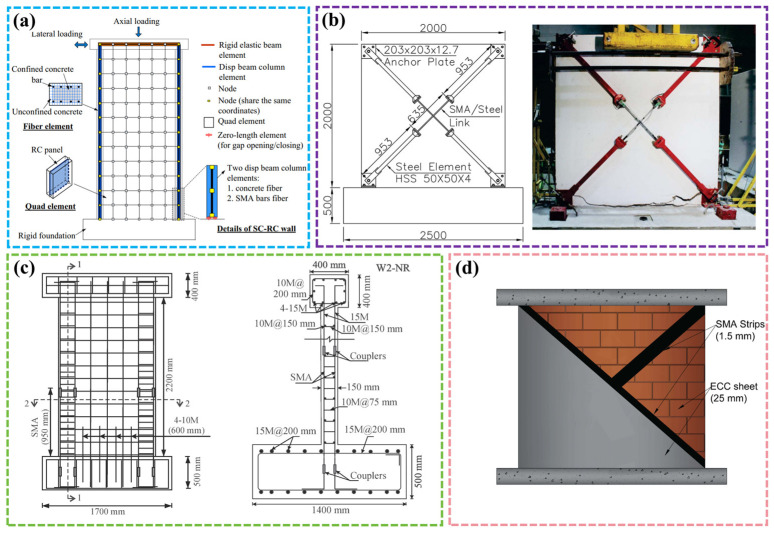
SMA-reinforced shear walls: (**a**) finite element modeling of plastic zone of SMA-reinforced shear wall [127]; (**b**) SMA externally reinforced shear wall [131,132,133]; (**c**) SMA-reinforced shear wall design [134]; (**d**) SMA-ECC shear wall [140].

The research of SMA-reinforced shear wall members mainly focuses on finite element analysis. Based on the shear wall test model proposed by Thomsen et al. [126], Wang and Zhu [127] replaced the steel bar in the edge-constrained key area with SMA and used the finite element software OpenSees to simulate and analyze the self-resetting performance of SMA shear walls, as shown in Figure 14a. The analysis results showed that the residual displacement of the SMA shear wall was still small even at 2.5% inter-story displacement. SMA could meet the seismic response of the structure under large earthquake conditions. Ghassemieh et al. [128,129] developed the SMA constitutive model using the Fortran language and embedded it into Abaqus finite element software. Based on this model, they analyzed the seismic response of a five-story coupled shear wall and a two-story shear wall structure and compared the experimental results to verify the effectiveness of the model. Abraik and Youssef [130] used the vulnerability curve to evaluate the seismic performance and vulnerability of 10-story and 20-story structures. The results of interlayer displacement, residual displacement, and vulnerability proved that even if SMA was only applied to the key parts of the edge constraint, it significantly improved the seismic performance and reduced the residual displacement of the structure.

Cortés-Puentes and Palermo [131,132,133] developed an SMA tension bracket, as shown in Figure 14b. Through experiments and simulations, it was proven that this device improved the strength of RC shear walls while reducing the strength and stiffness degradation caused by shear failure. Abdulridha and Palermo [134] designed an SMA-reinforced shear wall component, as shown in Figure 14c. Through the cyclic loading test, it was found that SMA significantly improved the crack recovery ability of the component, even if the residual crack was 12%. The energy consumption of the SMA shear wall was lower than that of the ordinary shear wall due to the SE SMA reinforcement. Subsequently, Cortés-Puentes et al. [135] replaced the concrete in the damaged area of an SMA shear wall and carried out a cyclic loading test on it. The results showed that the yield load and ultimate load of the repaired shear wall were consistent with the original shear wall, which proved that the SMA shear wall could continue to be used after the earthquake with simple repair. Soares et al. [136] evaluated the seismic performance of the SMA shear wall through numerical simulation. Compared with the traditional steel-reinforced wall, the SMA shear wall had better self-resetting and energy dissipation capabilities.

Kian and Cruz-Noguez [137] introduced three new types of shear walls: the FRP shear wall, steel wire shear wall, and SMA shear wall. The seismic performance of the three types of shear walls was compared through quasi-static loading tests, and the recoverability mechanism of shear walls was discussed. Later, Abraik and Assaf [138] compared the seismic performance of different types of SMA (NiTi, FeNCATB, and CuAlMn)-reinforced shear walls with different ground motion durations, and the results showed that Cu-based RC walls showed better performance. In addition, they [139] used slotting technology to realize an SMA-reinforced shear wall in 2022 and explored its seismic performance under uniaxial and cyclic loads. The results showed that this method improved the bearing capacity and ductility of the wall and delayed strength degradation. In order to solve the problem of brittle cracking of ordinary concrete, Tabrizikahou et al. [140] combined Ni-Ti SMA strips and ECC to form an SMA-ECC shear wall and used Abaqus software (https://www.3ds.com/products/simulia/abaqus) for evaluation, as shown in Figure 14d. The results revealed that the combination of the two methods improved the shear wall’s energy dissipation capacity and led to a superior self-resetting ability and self-healing ability, thereby improving the seismic performance and durability of the shear wall structure.

In summary, the SE SMA can indeed improve the recoverable deformation performance of the concrete shear wall members and effectively reduce the damage to the building structure in the event of an earthquake. At present, the production cost of SMA material is high. When designing and optimizing SMA shear walls, unique design methods and reasonable reinforcement should be adopted to maximize the SE characteristics of SMA with low cost. Therefore, it is necessary to further optimize the design of the SMA shear wall structure.

After investigation, few studies on SMA-reinforced slab members were found. Yeon et al. [141] conducted a flexural test on unidirectional RC slabs with Fe-SMA reinforcement as the tensile steel bars and evaluated the influence of the recovery stress of Fe-SMA reinforcement on the flexural performance of the slabs. The results showed that the number and width of bending cracks decreased with Fe-SMA reinforcement, which was mainly due to the prestressing effect of the recovery stress generated by the Fe-SMA reinforcement.

## 4. SMA Isolation Devices in Building Structures

Earthquakes are sudden, unpredictable, and highly harmful natural disasters. The casualties and economic losses caused by earthquakes have a great impact on society and the economy. The consequences of earthquakes cannot be ignored [142]. In China’s seismic design code [143], it is required that structures have sufficient stiffness and strength to resist earthquakes. However, the current methods dissipate seismic energy via the structure itself, which has a significant effect on the main body of the structure. Moreover, due to the uncertainty of the earthquake, the structure cannot adapt to the earthquake effect at various intensities. Such a building is very likely to collapse because it does not meet the safety and stability requirements. Due to its excellent SME and SE characteristics, SMA is mainly used in structural supports and dampers in building structures, and a considerable amount of associated research has been carried out.

### 4.1. SMA Bearings

The bearing of the structure is an important component connecting the structure and the foundation. It is responsible for the functions of transmitting load and shock isolation. Existing research focuses on the composite bearing combining SMA and rubber materials: the SMA-laminated rubber bearing. Introducing SMA material into ordinary laminated rubber bearings can enhance their durability and shock absorption performance. SMA laminated rubber bearings utilize the SE and SME of SMA to provide greater vertical tensile strength and horizontal damping capacity, thereby enhancing the stability and damping effect of the bearings under earthquake or other vibration conditions.

**Figure 15:**
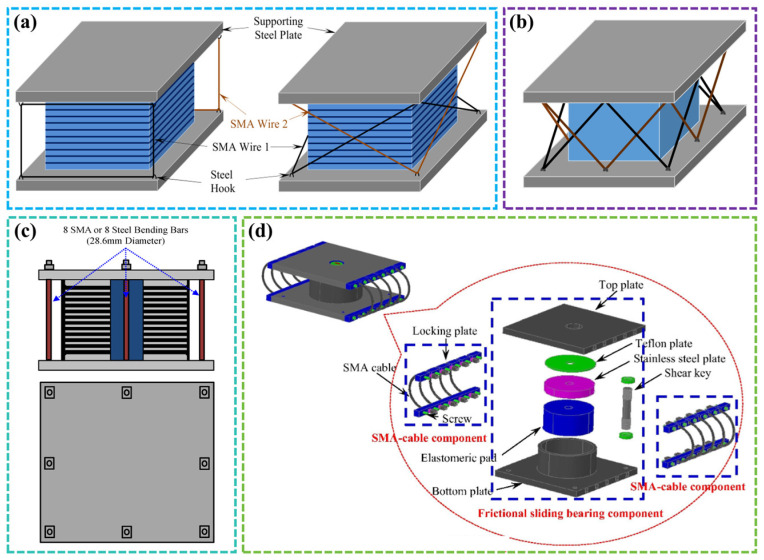
SMA bearings: (**a**) SMA-laminated rubber bearings with straight and cross arrangements [145]; (**b**) SMA-laminated rubber bearings with double cross arrangement [146]; (**c**) schematic diagram of LRB model with SMA bending rod [148]; (**d**) SMA friction sliding bearing [149].

Gjorgjiev and Garevski [144] combined low-damping rubber bearings with SMA as elastic elements, while Dezfuli and Alam [145] developed SMA-laminated rubber bearings with linear and cross arrangements, as shown in Figure 15a. In 2015, they [146] also studied double-cross SMA wires around the bearings, as shown in Figure 15b. Through numerical simulation, it was found that the presence of SMA wires improved the energy dissipation capacity to varying degrees, and the prestressing of SMA could enhance the vibration isolation capacity of SMA bearings. Li et al. [147] presented a new type of bearing: the SMA-wire-based roller bearing. Through experimental research, the results showed that the NiTi SMA prevented excessive displacement of the device, and the triangular constitutive model used accurately described the hysteresis behavior of the bearing.

SMA is also used in isolation bearings in other forms. Seo and Hu [148] installed SMA bending rods for self-centering on lead rubber bearings, as shown in Figure 15c. These bearings were equipped with different numbers of SMA bending rods and studied through nonlinear dynamic analysis. The results showed that the SMA bending rod system effectively reduced the residual displacement and provided greater flexibility and self-resetting ability for the entire building structure. Zheng et al. [149] developed and tested an isolation system composed of SMA stranded wire and friction sliding bearings, as shown in Figure 15d. A friction sliding bearing was used as the energy dissipation device, and the SMA stranded wire provided the reset ability. The test results revealed that the isolation system had a good energy dissipation and self-resetting effect. The related benefits of the earthquake-isolated building structure with SMA support could be quantified based on the assessment of toughness and life cycle loss. Cao et al. [150] designed a multi-level SMA/lead rubber bearing (ML-SLRB) and established and analyzed the finite element model of the system to evaluate its different responses in varying stages. The results indicated that the device could achieve multi-level performance and had lower residual displacement compared to traditional methods.

To sum up, research on SMA bearings mostly focuses on numerical simulation. Although there are relevant experimental data to assist in verifying the accuracy of the experiment, there is a lack of experimental data on the bearing entity. Static and shaking table tests on the bearing entity and further research and optimization are essential to understand the actual mechanical properties of the bearing and to lay a solid foundation for its practical application in engineering. Secondly, due to the high price of SMA, it is necessary to further consider the arrangement and connection mode when SMA is used with the bearing to give full play to the characteristics of SMA and enable the bearing to play a comprehensive and smart role.

### 4.2. SMA Dampers

A damper is a commonly used energy dissipation device that can effectively reduce the effects of earthquakes on structures. Combining a damper with SMA can not only improve the energy dissipation capacity but also provide a restoring force to better protect the damper, giving it the dual advantages of self-resetting and energy dissipation.

**Figure 16:**
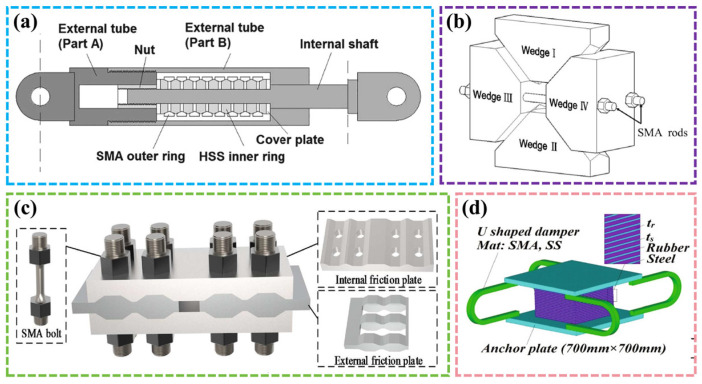
Various SMA dampers: (**a**) self-resetting damper [151]; (**b**) wedge damper [152]; (**c**) SMA-based variable friction damper [153]; (**d**) U-shaped damper [154].

Wang et al. [151] discussed the manufacturing process and mechanical properties of a new self-resetting damper with a core component of an SMA ring spring, as shown in Figure 16a. The experimental study found that the damper had good energy dissipation performance with an equivalent viscous damping ratio of up to 18.5%. It effectively resisted strong earthquakes and had self-resetting driving effect without requiring maintenance. Zhang et al. [152] and Chen et al. [153] designed a new type of SMA rod damper by combining SMA rod and a friction damper, as shown in Figure 16b,c, and conducted cyclic tensile and compressive tests and finite element simulation. It was found that the damper had a better energy dissipation ability than the damper with SMA wire and was more flexible. Its initial stiffness and deformation capacity could also be adjusted. Sheikhi et al. [154] studied the performance of a natural rubber bearing system (NRB) equipped with a U-shaped damper, as shown in Figure 16d. The results showed that the U-shaped SMA damper had SE behavior and residual deformation reversibility, and the finite element method could accurately simulate the mechanical behavior of the damper. After verifying the validity of the model, the optimal SMA-to-steel thickness ratio was obtained to maximize the energy consumption of the damper and minimize the residual deformation.

Asgarian et al. [155] applied a new SMA self-centering hybrid damper to a five-story frame structure for nonlinear time-history analysis and showed that the damper effectively controlled the acceleration and interlayer displacement of the structure. Mirzai et al. [156] proposed a smart damper design parameter optimization adjustment method based on the cuckoo search algorithm. The cuckoo search algorithm (CSA) was used to study the optimal parameters of a new type of smart damper under the effect of an earthquake, considering the seismic response of four-story and nine-story buildings with seven pairs of ground motions. The results indicated that the CSA was suitable for determining the optimal parameters of shear polyurethane friction devices and SMA plate systems for buildings and considering seismic motion.

Although some research work on SMA dampers has been carried out both domestically and internationally, the research on the mechanical properties and damping effect of these types of smart dampers is still relatively limited. Although the effectiveness of the SMA damper has been proven via theory and experiments, this method also has shortcomings. The energy dissipation unit (SMA alone or with other energy dissipation materials) in the damper is active at the same time, and the working state of the energy dissipation unit cannot be adjusted according to the response of the structure under earthquake conditions. The performance of SMA is dependent on the strong seismic performance requirements of the structure. The energy dissipation capacity of SMA is usually not fully utilized during small and medium earthquakes; so, it is necessary to make full use of its value and continue to innovate to develop more rational and smarter SMA dampers.

## 5. Practical Engineering Applications of SMA

**Figure 17:**
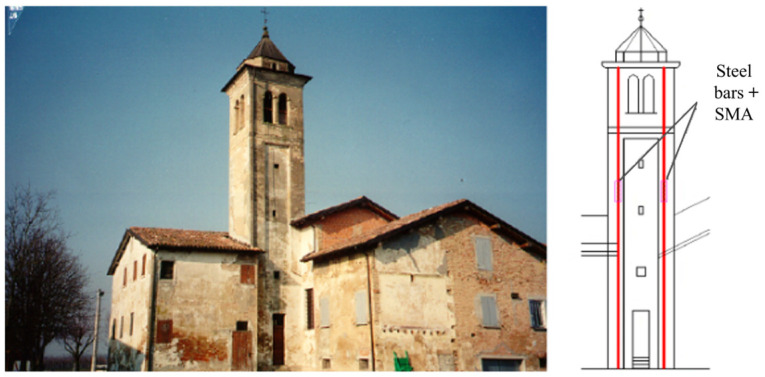
SMA application of San Giorgio Church in Italy [157].

The vibration control of building structures can be improved by SMA reinforcement. The bell tower of the Church of San Giorgio in Trignano, Italy, was damaged in the earthquake in October 1996. Italian engineers connected pre-tensile steel bars and Ni-Ti SMA bars in series and installed them at the four corners of the bell tower to increase the structure’s resistance to lateral dynamic loads [157], as shown in Figure 17. In the earthquake of 2000, the structure showed good hysteretic properties and was not damaged. Similar SMA-based reinforcement cases for ancient buildings are the Badia Fiorentina Bell Tower in Italy and the Sherith Israel building restoration project in San Francisco, USA [158]. They were not destroyed in subsequent earthquakes and showed good hysteretic properties.

**Figure 18:**
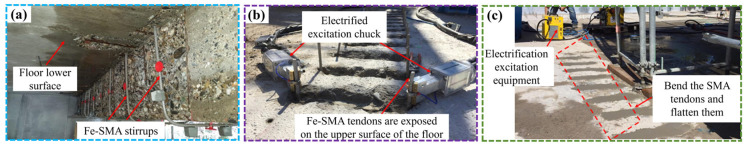
Process of active shear strengthening concrete beams with Fe-SMA stirrups: (**a**) installation and fixation of Fe-SMA stirrups; (**b**) energized excitation of Fe-SMA stirrups; (**c**) post-excitation trimming.

Based on reference [87], it has been proven that the combination of U-shaped Fe-SMA bars and sprayed cement mortar is effective for the shear reinforcement of T-shaped concrete beams. Shear reinforcement was carried out on the concrete beams of the renovated theater in Baden, Switzerland, with the operation steps shown in Figure 18. In addition to bending the exposed stirrups and repairing the groove after excitation, the strengthening procedure is consistent with that in the above study. The reinforcement project uses Fe-SMA stirrups to actively restrain the shear strength of concrete beams, a method which has no effect on the surface appearance of the structure after reinforcement. After the reinforcement is completed, it has no effect on the surface appearance of the structure. Moreover, because the prestress of Fe-SMA bars is generated via the metallography transformation of the material itself, there is no friction loss of prestress in the process.

**Figure 19:**
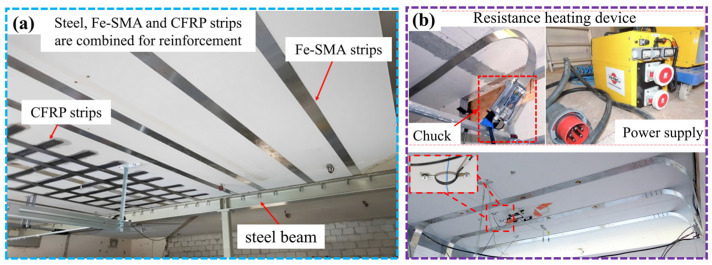
Fe-SMA-reinforced concrete slabs: (**a**) reinforcement example after removal of load-bearing wall [160]; (**b**) crack control of concrete slabs [160].

The Swiss company re-fer AG conducted the world’s first application of Fe-SMA strips in the field of building reinforcement in Villigen in 2017 [159]. Due to the removal of load-bearing walls, the static bearing capacity of the building’s reinforced concrete floor needed to be enhanced. By combining the use of steel beams, Fe-SMA strips and non-prestressed CFRP strips, the defects of insufficient configuration of bending steel bars at the location of the demolished wall are addressed, and the requirements of normal use and ultimate bearing capacity are met. The arrangement of reinforcement materials is shown in Figure 19a. The company also used nail fixation Fe-SMA strip technology to control cracks in the concrete slabs of a school building in Nieppe, France [160]. The Fe-SMA strip was fixed on the concrete slabs by nails, as shown in Figure 19b, and stimulated using a resistance heating device. The crack width was reduced by 0.14 mm after prestressing was generated.

## 6. Conclusions and Future Works

SMA has a significant effect on improving the mechanical properties of structural components of buildings and the smart deformation control ability of cracking self-recovery. SMA-reinforced structural components can give the structure a deformation and displacement self-recovery ability; improve the working performance; and achieve satisfactory results in the application of concrete beams, columns, beam–column joints, shear walls, and slabs. With the development of SMA material research and production technology, improving the comprehensive performance of SMA while reducing costs will help promote SMA-reinforced building structures as engineering materials and promote the large-scale development of smart concrete structures.

The main conclusions from summarizing and analyzing the research of SMA in building structures are as follows:

(1) With its unique SME and SE characteristics, SMA can achieve deformation self-recovery. Different types of SMA have different recovery performance, among which NiTi-based SMA has strong comprehensive performance and a wide range of applications. It is the most common material in building structure research, but its price is relatively high.
(2) The effect of SMA on enhancing the mechanical properties of structural components of buildings is affected by various factors, including the diameter and reinforcement ratio of SMA prestress value, tension method, and excitation method. With different loading modes, SMA enhances the self-healing, flexural, and shear properties of beam components, as well as the deformation energy dissipation capacity of column components, beam–column joints, and shear walls.
(3) Because of its excellent SME and SE characteristics, SMA is used in bearings and dampers in building structures to improve their mechanical properties under earthquake conditions.

Further research in this field can be carried out as follows:

(1) The construction technology standards regarding heating in SMA activation are not yet clear. Although excessive heat can be applied to ensure sufficient activation, in concrete components, excessive heat can hinder the formation of ettringite in the concrete matrix and cause longitudinal splitting cracks. The standardized SMA heating method needs to be improved.
(2) In order to overcome the shortcomings of brittleness and easy cracking of traditional concrete, a strong combination of SMA and ECC or UHPC should be established to study the deformation self-recovery ability of SMA-ECC/SMA-UHPC composite material components.
(3) SMA is relatively expensive. Considering the actual situation of the building structure, it is necessary to effectively combine SMA with other composite materials (such as CERP) to achieve their complementary advantages and enhance the performance and function of shape memory composite materials.
(4) The influence of factors such as prestressed tension and anchorage, the splicing mode, and the SME driving mode of SMA on the deformation recovery performance of SMA-reinforced structural components should be considered.
(5) Existing studies generally use wires with a small cross-sectional area as reinforcement materials. Few studies have been conducted using large-cross-section materials, and the relevant mechanical tests are limited to scale model components. In addition, the effects of state parameters such as temperature, pretension strain, and loading frequency on the SME of SMA still lack sufficient and effective data support. The nonlinear constitutive relationship of SMA in a complex stress state or multi-directional constraint state is still to be established.

## Data Availability

Not applicable.

## Appendix A

**Table A1:** Review of studies on SMA-reinforced RC beams (in chronological order).

Function	Study Year	Installation Technique	Property	Type of Loading	Research Method
Self-healing	Sakai et al. [61]-2003	Internal SMA reinforcement	SE	Three-point cyclic bending	Experimental
	Li et al. [64,65]-2007	Embedded Ni-Ti wires + CFRP plate	SME	Not reported	Experimental + Numerical
	Kuang and Ou [66]-2008	Internal Ni-Ti SMA wires + adhesives	SE	Three-point cyclic bending	Experimental
	Xue et al. [68]-2009	External installation of Ni-Ti SMA bars	SME	Four-point bending	Experimental
	Yang et al. [69]-2011	Internal/External installation of Ni-Ti SMA bars	SME	Three-point bending	Experimental
	Sun et al.-2011[70]; 2013 [71]	Internal/External installation of Ni-Ti SMA bars	SME	Three-point bending	Experimental
	Choi et al. [63]-2015	Internal Ni-Ti SMA fibers	SME	Three-point bending	Experimental
	Lee et al. [62]-2018	Internal Ni-Ti/Ni-Ti-Nb SMA fibers	SME	Three-point bending	Experimental
	Bonilla et al. [67]-2018	Internal Ni-Ti SMA wires + microcapsules	SME	Three-point bending	Experimental
Flexural behavior	Abdulridha et al. [81]-2013	Ni-Ti rebars as top and bottom longitudinal reinforcement at mid-span	SE	Four-point cyclic andreversed bending	Numerical
	Shahverdi et al. [72]-2016	Fe-SMA strips as NSM reinforcement	SME	Four-point bending	Experimental
	Shahverdi et al. [77]-2016	Fe-SMA rebars embedded in a shotcrete layer	SME	Four-point bending	Experimental
	Hong et al. [73]-2018	NSM Fe-SMA Strips	SME	Four-point bending	Experimental
	Rojob and El-Hacha [75]-2018	NSM Fe-SMA rebars	SME	Freeze–thaw cycles and sustained load	Experimental
	Rojob and El-Hacha [76]-2018	NSM Fe-SMA rebars	SME	Cycles of fatigue loading	Experimental
	Michels et al. [79]-2018	Externally anchored Fe-SMA strips	SME	Four-point bending	Experimental
	Strieder et al. [80]-2018	Externally anchored Fe-SMA strips	SME	Four-point bending	Experimental + Numerical
	Dolatabadi et al. [78]-2020	Fe-SMA rebars embedded in a shotcrete layer	SME	Four-point bending	Numerical
	Yeon et al. [74]-2021	Fe-SMA strips as NSM reinforcement	SME	Four-point bending	Numerical
	Hong et al. [82]-2022	Fe-SMA rebars as bottom longitudinal reinforcement	SME	Four-point bending	Experimental
	Yeon et al. [141]-2022	Fe-SMA rebars as tensile longitudinal reinforcement	SME	Four-point bending	Experimental
Shear behavior	Mas et al. [88]-2016	Externally installed Ni-Ti rectangular spirals	SE	Three-point cyclic bending	Experimental
	Montoya-Coronado et al. [83]-2019	Externally installed Fe-SMA strips as spirals	SME	Three-point bending	Experimental
	Cladera et al. [84]-2020	Externally installed Fe-SMA strips as spirals	SME	Three-point bending	Experimental
	Ruiz-Pinilla et al. [86]-2020	Externally installed Fe-SMA strips as spirals	SME	Three-point bending	Numerical + Analytical
	Czaderski et al. [87]-2020	U-shaped ribbed bars + cement-based mortar	SME	Four-point bending	Experimental
	Ji et al. [89]-2022	Internal Fe-SMA stirrups	SME	Four-point bending	Experimental + Numerical
	Abdulkareem et al. [85]-2023	Externally installed U-shaped Fe-SMA strips as spirals	SME	Four-point bending	Experimental
	Hong et al. [90]-2023	Internal Fe-SMA stirrups	SME	Four-point bending	Experimental + Numerical

**Table A2:** Review of studies on SMA-reinforced RC columns (in chronological order).

Function	Study Year	Installation Technique	Property	Type of Loading	Research Method
Axial compression property	Shin and Andrawes [92,93]-2010	External NiTiNb SMA spirals	SME	Uniaxial compression test	Experimental
	Choi et al.-2010 [94,95], 2011 [96]	External NiTiNb/NiTi SMA wires/steel jackets	SME	Uniaxial compression test	Experimental
	Chen et al. [100]-2014	External SMA wires + steel tubes	SME	Monotonic and cyclic loading	Numerical + Experimental
	Tran et al. [97]-2015	External NiTi SMA wires	SME	Uniaxial compression test	Experimental
	Chen and Andrawes [103]-2017	External NiTiNb SMA spirals	SME	Uniaxial cyclic loading	Experimental + Analytical
	Chen and Andrawes [104]-2017	External NiTiNb SMA spirals	SME	Monotonic and cyclic loading	Analytical + Numerical
	Gholampour and Ozbakkaloglu [108]-2018	External Ni-Ti SMA spirals	SME	Uniaxial compressive test	Experimental
	Hong et al. [99]-2020	External NiTi SMA wires	SE	Uniaxial compressive test	Experimental + Analytical
	Suhail et al. [101]-2020	External NiTiNb SMA spirals and FRP systems	SME	Uniaxial compressive test	Experimental
	El-Hacha and Abdelrahman [102]-2020	External Ni-Ti SMA spirals	SME	Uniaxial compressive test	Experimental
	Khaled and El-Hacha [105]-2020	External SMA spirals	SME	Uniaxial compressive test	Analytical
	Jeong et al. [106]-2022	External Fe-SMA strips	SME	Uniaxial compressive test	Experimental
	Zerbe et al. [108]-2022	External Fe-SMA strips	SME	Uniaxial compressive test	Experimental + Analytical
	Vieira et al. [109]-2022	External Fe-SMA strips	SME	Uniaxial compressive test	Analytical + Numerical
	Han et al. [107]-2023	External Fe-SMA strips + FRP	SME	Uniaxial compressive test	Experimental + Analytical
Seismic performance	Shin and Andrawes [112]-2011	External SMA spirals and FRP wraps	SME	Quasi-static lateral cyclic loading	Experimental
	Shin and Andrawes [113]-2011	External SMA spirals	SME	Quasi-static lateral cyclic loading	Experimental

**Table A3:** Review of studies on SMA-reinforced RC beam–column joints (in chronological order).

Function	Study Year	Installation Technique	Property	Type of Loading	Research Method
Seismic performance	Youssef et al. [114]-2008	Ni-Ti SMA internal reinforcement in the plastic hinge region	SE	Quasi-static reversed cyclic loading	Experimental
	Alam et al. [115]-2008	Ni-Ti SMA internal reinforcement in the plastic hinge region	SE	Quasi-static reversed cyclic loading	Analytical + Numerical
	Alam et al. [116]-2009	Ni-Ti SMA internal reinforcement in the plastic hinge region	SE	Quasi-static reversed cyclic loading	Experimental + Numerical
	Zafar and Andrawes [121]-2015	SMA internal reinforcement in the plastic hinge region + FRP	SE	Quasi-static reversed cyclic loading	Numerical + Analytical
	Oudah and El-Hacha [118]-2017	Ni-Ti SMA internal reinforcement anchored using screw lock steel anchors	SE	Quasi-static cyclic loading	Experimental + Analytical
	Oudah and El-Hacha [119]-2018	Ni-Ti SMA internal reinforcement in the plastic hinge region	SE	Quasi-static reversed cyclic loading	Experimental
	Nahar et al. [117]-2019	Ni-Ti SMA internal reinforcement in the plastic hinge region	SE	Quasi-static reversed cyclic loading	Numerical + Analytical
	Navarro-Gómez and Bonet [122]-2019	SMA bars and ultra-high-performance concrete in the plastic hinge region	SE	Quasi-static cyclic loading	Numerical
	Pei et al. [120]-2022	SMA bars and a steel plate in the core area of the joint	SE	Low cyclic loading test	Experimental + Numerical
	Qian et al. [123]-2022	ECC and SMA bars in the plastic hinge region	SE	Quasi-static cyclic loading	Experimental
	Qian et al. [124]-2022	ECC and SMA bars in the plastic hinge region	SE	Quasi-static reversed cyclic loading	Experimental + Numerical
	Qian et al. [125]-2023	ECC and SMA bars in the plastic hinge region	SE	Quasi-static reversed cyclic loading	Experimental

**Table A4:** Review of studies on SMA-reinforced RC shear walls (in chronological order).

Function	Study Year	Installation Technique	Property	Type of Loading	Research Method
Seismic performance	Ghassemieh et al.-2012 [128]; 2017 [129]	Diagonal SMAs in coupling beams	SE	Quasi-static reversed cyclic loading	Numerical
	Abdulridha and Palermo [134]-2017	Ni-Ti SMA rebars as internal longitudinal reinforcement in the plastic hinge regions	SE	Quasi-static reversed cyclic loading	Experimental
	Wang and Zhu [127]-2018	Ni-Ti bars in the plastic hinge regions	SE	Quasi-static reversed cyclic loading	Numerical
	Abraik and Youssef [130]-2018	SE-SMA bars in the plastic-hinge regions	SE	Quasi-static cyclic loading	Numerical
	Navarro-Gómez and Palermo-2017 [133]; 2018 [132]	Ni-Ti SMA rebars as external braces	SE	Quasi-static reversed cyclic loading	Experimental + Numerical
	Kian and Cruz-Noguez [137]-2018	Ni-Ti SMA internal reinforcement in the boundary element	SE	Quasi-static reversed cyclic loading	Experimental
	Navarro-Gómez and Palermo [131]-2020	Ni-Ti SMA rebars as external braces	SE	Quasi-static reversed cyclic loading	Numerical
	Córtes-Puentes et al. [135]-2018	Ni-Ti SMA internal reinforcement in the plastic hinge region	SE	Quasi-static cyclic loading	Experimental
	Soares et al. [136]-2021	Ni-Ti SMA internal reinforcement in the plastic hinge region	SE	Quasi-static reversed cyclic loading	Numerical
	Abraik and Assaf [138]-2021	Three different types of SE-SMA in the plastic hinge	SE	Quasi-static cyclic loading	Numerical
	Abraik and Ateeyah [139]-2022	SMA bars replacing the conventional steel bars located in the wall boundaries	SE	Quasi-static reversed cyclic loading	Numerical
	Tabrizikahou et al. [140]-2022	Ni-Ti strips and ECC sheets	SE	Cyclical lateral loading	Numerical
